# The effect of carbetocin compared to misoprostol in management of the third stage of labor and prevention of postpartum hemorrhage: a systematic review

**DOI:** 10.1186/s13643-018-0832-4

**Published:** 2018-10-20

**Authors:** Mohamed A. Abd El Aziz, Ahmed Iraqi, Parvin Abedi, Shayesteh Jahanfar

**Affiliations:** 1 Primary Health Care Physician, Ministry of Health, Al Qaliobia, Egypt; 2 Cairo University Hospitals, Giza, Egypt; 3 Midwifery Department, Nursing and Midwifery School, Menopause Andropause Research Center, Ahvaz Jundishapur University of Medical Sciences, Golestan Ave, Ahvaz, Iran; 4 School of Health Sciences, Health Professions 2239, Central Michigan University, Mount Pleasant, MI USA

**Keywords:** Carbetocin, Misoprostol, Postpartum hemorrhage, Third stage of labor

## Abstract

**Background:**

Postpartum hemorrhage (PPH) and the amount of blood loss are directly related to management of the third stage of labor. No previous report has compared the effects of carbetocin to those of misoprostol. The aim of this systematic review was to compare the effects of carbetocin to those of misoprostol for management of the third stage of labor and for the prevention of PPH.

**Methods:**

We searched the Cochrane Library (Central), Web of Science, Scopus, Science Direct, Ovid, clinicaltrial.gov, and PubMed databases on December 28, 2017. Data extraction and risk of bias assessment were performed by 2 of the authors independently. Individual and pooled incidences were calculated for the included studies, with 95% confidence intervals (CIs). We used a fixed model for forest plots without heterogeneity and a random effect model for those with heterogeneity.

**Results:**

Our search identified 117 studies; however, 29 studies were duplicate. Of the 88 non-duplicate studies, 5 met the inclusion criteria. Of these five studies, two are currently underway. Hence, three studies were finally included in our meta-analysis. The pooled estimate of the impact of carbetocin on PPH (500–1000 ml) was (OR 0.27, 95% CI 0.14–0.50). Carbetocin significantly reduced the need for additional uterotonics (RR 0.28, 95% CI 0.15 to 0.49). Reduction in the hemoglobin level and blood loss during the third stage of labor was significantly lower in women who received carbetocin than in those who received misoprostol. The length of the third stage of labor was significantly lower in women who received carbetocin than in those who received misoprostol. The incidence of side effects, such as heat sensation, metallic taste, fever, and shivering, were significantly lower in women who received carbetocin than in those who received misoprostol.

**Conclusion:**

Although this review showed that carbetocin is effective for decreasing PPH, blood loss, the length of the third stage of labor, and the need for additional uterotonics, this conclusion should be considered with caution. Because assessment of PPH is a subjective issue and it is uncertain whether outcomes were assessed blindly in respect to treatment. We recommend future research to verify our findings. Also clinicians may like to consider use of carbetocin for women with low risk for PPH.

**Electronic supplementary material:**

The online version of this article (10.1186/s13643-018-0832-4) contains supplementary material, which is available to authorized users.

## Background

Postpartum hemorrhage (PPH) is defined as blood loss of > 500 ml within 24 h after normal vaginal delivery or > 1000 ml after cesarean section [1]. PPH and the amount of blood loss are directly related to management of the third stage of labor. The prevalences of PPH in developed countries have been reported to be 5% and 13% with active management and expectant management, respectively, during labor in vaginal delivery [2]. However, over the last few decades the frequencies of PPH of > 1000 ml have increased to 1% and 3% with active management and expectant management, respectively in the third stage of labor [2].

PPH is the leading cause of maternal mortality, and it has been estimated that 35% of maternal deaths are related to PPH [3]. From 1990 to 2010, there was a global reduction in the maternal mortality ratio (MMR) from 400 to 210 per 100,000 live births. However, the MMR has been shown to be higher in developing countries than in developed countries (240 vs. 16 per 100,000 live births) [4]. It has been found that most maternal deaths due to PPH (around 99%) are occurring in developing countries [5].

The World Health Organization recommends active management in the third stage of labor, and uterotonics such as oxytocin (10 IU, intramuscular/intravenous), should be administered for the prevention of PPH in all women who have given birth [6]. Various types of medications have been assessed. A previous study showed that administration of uterotonics (oxytocin or methylergometrine) immediately after expulsion of the fetal anterior shoulder can significantly reduce the occurrence of PPH of > 500 ml when compared to the occurrence with administration of uterotonics after expulsion of the placenta [7].

Administration of 800 μg of misoprostol, which is equivalent to 40 IU of intravenous oxytocin, can prevent PPH, and this approach can be used in the treatment of PPH [8]. Additionally, there is evidence supporting the use of 600 μg of misoprostol sublingually by skilled or non-skilled caregivers in developing countries, which carries the same effect as that of 800 μg of misoprostol [9–11].

Carbetocin is an oxytocin agonist that has uterotonic effects for the prevention of PPH. A Cochrane systematic review of 11 studies (2635 women) showed that carbetocin could significantly reduce the risk of PPH when compared to the risk with oxytocin in women who underwent cesarean section (risk ratio 0.55, 95% confidence interval [CI] 0.31–0.95) [12]. A recent study showed that carbetocin could significantly reduce the occurrence of PPH after cesarean section when compared to the occurrence with placebo, but carbetocin could not significantly reduce the occurrence of PPH after normal vaginal delivery [13]. Canadian Society of Obstetricians and Gynecologists (SOGC) in their new guideline for active management of the third stage of labor, recommended the use of carbetocin (100 μg) as an IV bolus over 1 min for prevention of PPH in elective cesarean section and normal vaginal delivery in women who have a one risk factor for PPH instead of oxytocin [14]. Also Leung et al., in their study on 329 women who gave birth normally found that carbetocin has a comparable effect to syntometrine in terms of hemoglobin reduction, PPH, additional need for oxytocin and retained placenta with lesser side effects such as nausea, vomiting, and hypertension [15].

Some studies have compared the effects of carbetocin with those of placebo or oxytocin, but there is no report comparing the effects of carbetocin to those of misoprostol. This systematic review aimed to compare the effects of carbetocin to those of misoprostol for management of the third stage of labor and for the prevention of PPH.

## Methods

This systematic review considered randomized controlled trials, and quasi-experimental studies in which carbetocin was compared with misoprostol. This review was performed in accordance with the Preferred Reporting Items for Systematic Reviews and Meta-Analyses (PRISMA, Appendix No:1) standard [16].

Studies that met the following criteria were included (1) women underwent vaginal or caesarian delivery with or without risk factors for PPH [17]; 2) carbetocin was compared with misoprostol (any route of administration and any dose).

### Types of outcomes

#### Primary outcomes

The primary outcomes were PPH (> 500 ml blood loss), severe bleeding (> 1000 ml blood loss), in need for additional uterotonics, and need for blood transfusion.

#### Secondary outcomes

The secondary outcomes were need for additional interventions within 24 h after childbirth, need for manual removal of the placenta, need for intensive care unit (ICU) admission within 24 h after childbirth, maternal death, decrease in the hemoglobin level, blood pressure change at delivery and then 1 h postpartum, duration of the third stage of labor (time needed for expulsion of the placenta) > 30 min, length of hospital stay > 24 h for normal vaginal delivery and > 72 h for cesarean section, heart rate change at delivery and then 1 h postpartum, and side effects from hemorrhage or PPH treatment (e.g., headache, heat sensation, abdominal pain, palpitations, metallic taste, fever, shivering, nausea, vomiting, and pruritus).

Other core outcomes for PPH prevention such as shock, women’s sense of well-being, women’s satisfaction with intervention, breastfeeding, and also other core outcomes for PPH treatment such as coagulopathy, hysterectomy, and organ dysfunction [18] were not assessed in the review.

### Search strategy

The following databases were searched from inception on December 28, 2017, without any language or time restriction:

1. The Cochrane Central Register of Controlled Trials (CENTRAL) and the Cochrane Library 2017. (Cochrane search terms in Appendix No. 2) were used;
2. Web of Science (all databases; Web of Science terms in Appendix No. 2 were used).
3. Scopus (Scopus terms in Appendix No. 2 were used);
4. Science Direct; (Science Direct terms in Appendix No. 2 were used);
5. Ovid Medline (Ovid terms in Appendix No. 2 were used);
6. ClinicalTrial.gov (Clinicaltrial.gov terms in Appendix No. 2 were used); and
7. PubMed (PubMed terms in Appendix No. 2 were used). The search strategies for these databases are presented in Appendix No. 2.

All references of the articles were checked to identify any unknown trials that were not indexed in the searched databases.

### Selection of studies

Two reviewers (MAA and AI) independently screened the title and abstract of each study that met the inclusion criteria. Disagreements were resolved through discussion.

### Data extraction

A data extraction form was designed for this study (Appendix 3). Two authors (MAA and AI) extracted the data independently, and discrepancies were resolved through discussion. Data were then entered into Review Manager (RevMan 5.3) for data analysis and were checked for accuracy. Data extraction sheet was included assessment of quality, demographic data, study design, primary, and secondary outcomes.

### Assessment of the risk of bias in the included studies

Two reviewers (MAA and AI) assessed the risk of bias independently. Disagreements were resolved through discussion. We used the criteria outlined in the Cochrane handbook for Systematic Reviews of Interventions [19] as follow: random sequence generation (selection bias), allocation concealment (selection bias), blinding of the participants and the personnel (performance bias), blinding of outcome assessment (detection bias), incomplete outcome data (attrition bias), selective reporting (reporting bias), and other risks of bias (Fig. 2).

### Statistical analyses

Review Manager (RevMan 5.3) software was used to analyze the data. Individual and pooled incidences were calculated for the included studies with 95% CI. For studies without heterogeneity, a fixed model was used, while for studies with heterogeneity, a random model was used. Statistical heterogeneity was evaluated using the chi-square test and if *I*^2^ was greater than 20%, the random model was used. The RR with 95% CI was calculated for dichotomous data, while mean differences (MDs) were calculated for continuous data. A *p* value < 0.05 was considered significant.

## Results

**Fig. 1:**
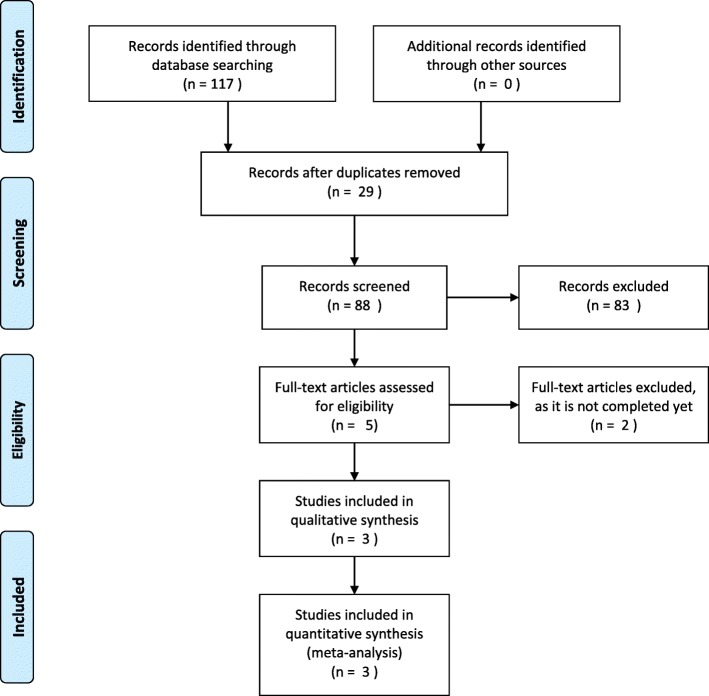
PRISMA flow diagram

Our search identified 117 studies; however, 29 studies were duplicate. Of the 88 non-duplicate studies, 83 did not meet the inclusion criteria and 5 met the inclusion criteria. Of the 5 studies, 2 are currently underway, and thus, 3 studies were finally included in our meta-analysis (Fig. 1). All studies recruited women at pre delivery stage.

The first study was a randomized controlled trial that was performed in Shebin-Elkom, Egypt [20], and it included 281 women in 3 groups (oxytocin, carbetocin, and misoprostol). They enrolled women with singleton fetus, and women who received routine active management of the third stage of labor. Women received one of the following regimens: intraumblical oxytocin, intravenous carbetocin 1 ml (100 mcg) or sublingual misoprostol (400 μg).

The second study also was a RCT that was performed in Cairo, Egypt [21], and it included 270 women in 3 groups (carbetocin [*n* = 90], misoprostol [*n* = 90], and oxytocin [*n* = 90]). Women with singleton baby and full-term pregnancy enrolled in this study. Women after vaginal delivery or cesarean section received either 1 ml (100 μg) carbetocin by infusion, or two sublingual misoprostol (each 200 μg) or 10 IU/ml oxytocin by infusion.

The third study (RCT) was performed in Benha, Egypt [22] by Mohamad Ibrahim and it included 60 severe pre-eclamptic patients in 2 groups (carbetocin [*n* = 30] and misoprostol [*n* = 30]). Women with severe preeclampsia and singleton baby, gestational age > 28 weeks and vaginal delivery were included in this study. Women in the carbetocin group received 100 μg carbetocin by slow intravenous bolus, and the other group received 600 μg misoprostol sublingually after delivery of the baby.

**Fig. 2:**
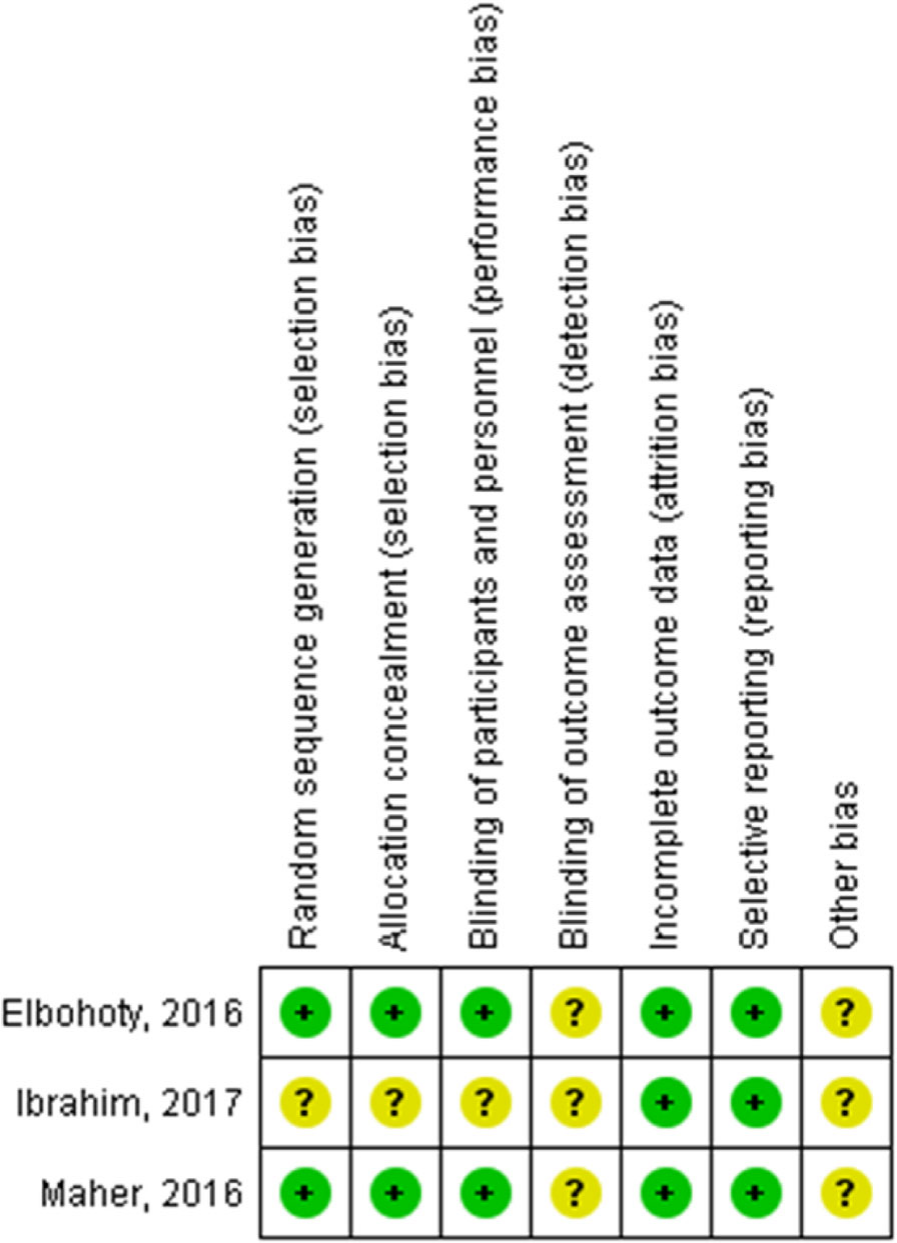
Risk of bias in included studies

The risk of bias for the included studies is presented in Fig. 2. As evident from this figure, Maher et al. and Elbohoty et al’s studies were low risk of bias for random sequence generation, allocation concealment, and blinding of participants, while Ibrahim et al’s study was unclear risk for the above-mentioned issues. All three studies were unclear risk of bias for blinding of outcome assessment and low risk of bias for incomplete outcome data and selective reporting and unclear risk of bias for “other biases.” Also all studies scored “low risk of bias” for publication bias.

**Fig. 3:**
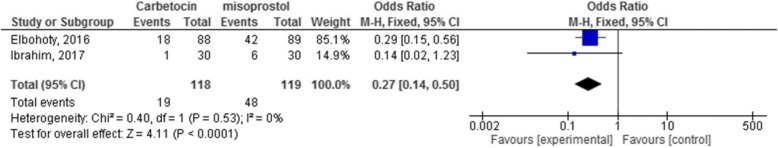
Forest plot of pooled estimated incidence of PPH (95% of confidence interval)

The pooled estimate of the impact of carbetocin on PPH (500–1000 ml) was (OR 0.27, CI 0.14–0.50), study 21and 22, 237 participants) (Fig. 3).

**Fig. 4:**
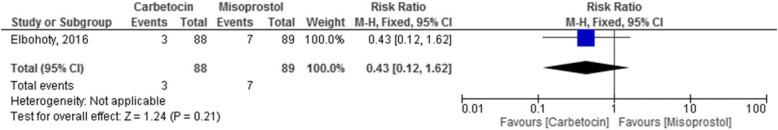
Forest plot of pooled estimated of severe PPH (> 1000 ml) (95% confidence interval)

There was no adequate evidence for the effectiveness of carbetocin in reducing severe PPH (> 1000 ml) when compared with misoprostol. Only one study performed this comparison, and there was no significant difference in the reduction of the risk of severe PPH when women who received carbetocin and those who received misoprostol were compared (RR 0.43, 95% CI 0.12 to 1.62, study 21, 177 participants) (Fig. 4).

**Fig. 5:**
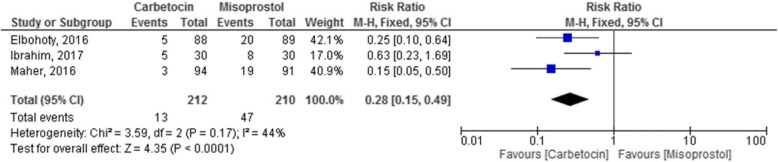
Additional need for uterotonics in carbetocin and misoprostol groups (95% confidence interval)

Carbetocin significantly reduced the need for additional uterotonics (RR 0.28, 95% CI 0.15 to 0.49, studies 20–22, 422 participants) in women who underwent cesarean section and those who underwent normal vaginal delivery (Fig. 5). In vaginal deliveries, the heterogeneity was high (*I*^2^ = 63%). When we excluded Maher et al’s study that was the reason for heterogeneity, only one study remained (Ibrahim et al). Because of few number of studies, we were unable to do subgroup analysis.

Need for blood transfusion and manual removal of the placenta were not significantly different between women who received carbetocin and those who received misoprostol, irrespective of whether the women had cesarean section or vaginal delivery (RR 0.57, 95% CI 0.21–1.58, study 21, 422 participants), (RR 0.73, 95% 0.48 to 1.1, studies 20, 22, 245 participants) respectively. There was no maternal death or ICU admission in the three studies [20–22].

Reduction in the hemoglobin level was significantly lower in women who received carbetocin than in those who received misoprostol among women who underwent cesarean section (MD − 4.00, 95% CI − 4.83 to − 3.11, *p* = 0.00001, study 21, 177 participants), and vaginal delivery (MD − 0.11, 95% CI − 0.14 to − 0.07, studies 20, 22, 245 participants). The heterogeniety in the vaginal delivery was high (*I*^2^ = 99%) that shows may we should not mix the studies together.

**Fig. 6:**
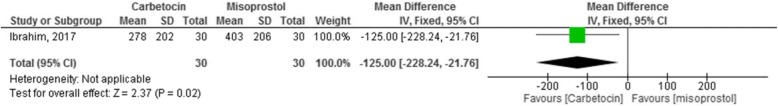
Mean of blood loss in normal vaginal delivery in two groups of carbetocin and misoprostol

**Fig. 7:**
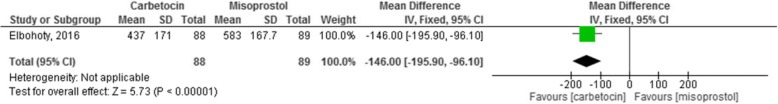
Mean of blood loss in cesarean section in two groups of carbetocin and misoprostol

Blood loss in the third stage of labor and in the postpartum period was significantly lower in women who received carbetocin than in those who received misoprostol among women who had normal vaginal delivery (MD − 125, 95% CI − 228.2 to − 21.7, study 22, 60 participants) (Fig. 6) and those who underwent cesarean section (MD − 146, 95% CI − 195.9 to − 96, study 21, 177 participants) (Fig. 7).

The mean systolic blood pressure was significantly lower in women who received carbetocin than in those who received misoprostol (MD − 3.30, 95% CI − 4.48 to − 2.12, study 22, 60 participants); however, there was no significant difference in the mean diastolic blood pressure (MD − 0.10, 95% CI − 1.03 to 0.83, study 22, 60 participants).

The length of the third stage of labor was significantly lower in women who received carbetocin than in those who received misoprostol (MD − 4.70, 95% CI − 8.81to − 0.59, studies 20, 22, 245 participants). The mean length of hospital stay was not significantly different between women who received carbetocin and those who received misoprostol (MD 0.12, 95% CI − 0.03 to 0.27, 185 participants, study:20). However, the heart rate was significantly higher in women who received misoprostol than in those who received carbetocin (MD − 1.90, 95% CI − 3.47 to − 0.33, study 22, 60 participants).

With regard to side effects, the rates of heat sensation, metallic taste, fever, and shivering were significantly lower in women who received carbetocin than in those who received misoprostol [21, 22], while the rates of headache, abdominal pain, palpitation, nausea, vomiting [21, 22], and pruritus [21] were not significantly different between women who received carbetocin and those who received misoprostol.

## Discussion

This systematic review aimed to compare the effect of carbetocin with misoprostol for management of the third stage of labor and prevention of postpartum hemorrhage. Three studies (*n* = 422 women) included for meta-analysis in this study.

A previous study has shown that oxytocin is an important uterotonic agent that can decrease blood loss > 500 ml in the third stage of labor and reduce the risk of PPH [23]. Misoprostol is a prostaglandin E1 analogue that can be considered as an uterotonic agent, and it can be administered sublingually, orally, vaginally, or via the rectum [24]. Carbetocin is a long-lasting agonist of oxytocin, and it can cause tetanic and rhythmic contractions in the uterus [25].

The present study found that carbetocin could significantly reduce PPH (500–1000 ml) in normal vaginal delivery and cesarean section, but there was inadequate evidence for the effectiveness of carbetocin in reducing severe PPH (> 1000 ml). A study by Khalafalah et al. showed that administration of carbetocin could significantly reduce the amount of blood loss in the third stage of labor (carbetocin vs. oxytocin 366.4 ± 165 vs. 434.7 ± 191.7 ml, *p* = 0.01) [25].

Additionally, carbetocin significantly reduced the need for additional uterotonics in women who underwent cesarean section or normal vaginal delivery. Attilako et al. found that carbetocin could significantly reduce the need for additional uterotonics in comparison with oxytocin in women who underwent cesarean section (RR 0.74, 95% CI 0.57–0.95) [26]. These results are in line with our findings. Leduce et al. recommend that carbetocin can be used 100 μg as an IV bolus over 1 min, after elective cesarean section instead of oxytocin for reducing PPH. Also carbetocin can be used for women with one risk factor for prevention of PPH instead of oxytocin in normal vaginal deliveries [14].

Our results showed that the hemoglobin level was significantly lower in women who received misoprostol than in those who received carbetocin among women who underwent cesarean section. However, among women who had normal vaginal delivery, the hemoglobin was not significantly different between women who received carbetocin and those who received misoprostol. Jagielska et al. compared the effects of carbetocin and oxytocin on PPH after cesarean section. They found that although the reductions in the levels of hemoglobin and hematocrit 24 h after cesarean section were greater in women who received oxytocin than in those who received carbetocin, the difference was not significant (hemoglobin − 1.24 vs. 1.17 g/dl; hematocrit − 3.26 vs. 2.93%) [27]. Also, Leung et al., in their study on 329 women who randomized in two groups of carbetocin (100 μg IM) or ergometrine (0.5 mg IM), found that the need to additional uterotonic agents, postpartum hemorrhage, and retained placenta was similar in both groups. Except for maternal tachycardia, carbetocin significantly was associated with lower adverse effect such as nausea, vomiting, and hypertension [15]. These results are similar to our findings.

The present study showed that blood loss in the third stage of labor and in the postpartum period was significantly lower in women who received carbetocin than in those who received misoprostol among women who underwent normal vaginal delivery and those who underwent cesarean section. These results are consistent with the findings in the study by Khalafalah et al. (366.4 ± 165 vs. 434.7 ± 191.7 ml, *p* = 0.01) [25].

The length of the third stage of labor was significantly lower in women who received carbetocin than in those who received misoprostol. The mean length of hospital stay was not significantly different between women who received carbetocin and those who received misoprostol. Su et al. showed that the length of the third stage of delivery was not significantly different between women who received carbetocin and those who received syntometrine [28]. These results are not consistent with our findings. The discrepancy may be associated with the nature of uterotonics.

The heart rate was significantly higher in women who received misoprostol than in those who received carbetocin. Additionally, the rates of heat sensation, metallic taste, fever, and shivering were significantly lower in women who received carbetocin than in those who received misoprostol. Su et al. compared the effects of carbetocin to those of syntometrine and found that side effects, such as nausea, vomiting, tremor, and uterine pain, were more prevalent in women who received syntometrine than in those who received carbetocin [28]. The results of the study by Su et al. are consistent with our findings.

### Strengths and limitations of our study

To the best of our knowledge, this is the first systematic review to compare the effects of carbetocin to those of misoprostol. The methodology adopted for this review was in accordance with Cochrane systematic review methodology for international studies. Publication bias is a possibility in this review because of the limited number of studies (all studies were from Egypt) and the small sample sizes. The heterogeneity in some cases such as additional need for uterotonic, reduction in Hb level, duration of third stage of labor, need for additional uterotonic, abdominal pain, shivering, and fever was high (more than 50%). Because in all cases of heterogeneity, two studies were entered to the meta-analysis, we were not able to exclude one study. It is also worth noting that blood pressure measurement is a sensitive measure of bodily homodynamic status and unless it is repeatedly measured in several occasions with 15 min time lapse between and after the patient is fully rested in the lying position, the measurements are sketchy at best. This limitation may impact a clinical decision based on our results. Furthermore, the small number of women enrolled in three studies reveals the necessity of conducting other interventional studies in the future. And finally, although two studies in this systematic review enrolled women with low risk for PPH, the third study enrolled severe pre-eclamptic women (*n* = 60) that are at the higher risk for PPH.

## Conclusion

Although this review showed that carbetocin is effective for decreasing PPH, blood loss, the length of the third stage of labor, and the need for additional uterotonics, this conclusion should be considered with caution. Because assessment of PPH is a subjective issue and it is uncertain whether outcomes were assessed blindly in respect to treatment. We recommend future research to verify our findings. Also clinicians may like to consider use of carbetocin for women with low risk for PPH.

## Additional files

Additional file 1: Data extraction form and Quality assessment. (ZIP 17 kb)

## Abbreviations

CI: Confidence interval
ICU: Intensive care unit
MD: Mean difference
MMR: Maternal mortality rate
PPH: Postpartum hemorrhage
RR: Risk ratio
